# Myoelectrically controlled wrist robot for stroke rehabilitation

**DOI:** 10.1186/1743-0003-10-52

**Published:** 2013-06-10

**Authors:** Rong Song, Kai-yu Tong, Xiaoling Hu, Wei Zhou

**Affiliations:** 1 School of Engineering, Sun Yat-sen University, Guangzhou, Guang Dong, P.R China; 2 Interdisciplinary Division of Biomedical Engineering, The Hong Kong Polytechnic University, Hung Hom Kowloon, Hong Kong

## Abstract

**Background:**

Robot-assisted rehabilitation is an advanced new technology in stroke rehabilitation to provide intensive training. Post-stroke motor recovery depends on active rehabilitation by voluntary participation of patient’s paretic motor system as early as possible in order to promote reorganization of brain. However, voluntary residual motor efforts to the affected limb have not been involved enough in most robot-assisted rehabilitation for patients after stroke. The objective of this study is to evaluate the feasibility of robot-assisted rehabilitation using myoelectric control on upper limb motor recovery.

**Methods:**

In the present study, an exoskeleton-type rehabilitation robotic system was designed to provide voluntarily controlled assisted torque to the affected wrist. Voluntary intention was involved by using the residual surface electromyography (EMG) from flexor carpi radialis(FCR) and extensor carpi radialis (ECR)on the affected limb to control the mechanical assistance provided by the robotic system during wrist flexion and extension in a 20-session training. The system also applied constant resistant torque to the affected wrist during the training. Sixteen subjects after stroke had been recruited for evaluating the tracking performance and therapeutical effects of myoelectrically controlled robotic system.

**Results:**

With the myoelectrically-controlled assistive torque, stroke survivors could reach a larger range of motion with a significant decrease in the EMG signal from the agonist muscles. The stroke survivors could be trained in the unreached range with their voluntary residual EMG on the paretic side. After 20-session rehabilitation training, there was a non-significant increase in the range of motion and a significant decrease in the root mean square error (RMSE) between the actual wrist angle and target angle. Significant improvements also could be found in muscle strength and clinical scales.

**Conclusions:**

These results indicate that robot-aided therapy with voluntary participation of patient’s paretic motor system using myoelectric control might have positive effect on upper limb motor recovery.

## Background

Stroke is the first leading cause of motor disabilities in many countries, which will significantly affect the daily activities of stroke survivors. Rehabilitation training provides an opportunity to improve motor function. Conventionally, it can be conducted by therapists in a one-on-one manual mode in hospital. In the last two decades, many advanced rehabilitation technologies are extensively developed, which provide repetitive, well-controlled assistance for the patients and relieve therapist from labour-intensive work [1-4].

Mechanical design is important to decide the joint kinematics of the extremity in the robot-aided rehabilitation training. There are two types of rehabilitation robot from the mechanical design point of view: end-effector based robots, and exoskeleton-type robots. MIT-MANUS was an example to the end-effector based robots, which interacted with subjects at the end of robot arm [4]. The design of end-effector could adapt to subjects with different body size. While exoskeleton-type robots can resemble human anatomy and apply torque to specific joints, moreover, the working-space of the rehabilitation training provided by such kinds of exoskeletions could approximate the working-space performed by human subjects [2,3]. Control strategy is another important factor to affect the training effect of robot-aided rehabilitation. MIT-MANUS applied impedance control in the robot-assisted upper limb rehabilitation, and it could keep a compliant trajectory under perturbation and promote interaction between subject and robotic system [4]. Mirror-Image Movement Enabler (MIME) was another rehabilitation robot which could assist bilateral elbow and shoulder movements within a three-dimensional space [5]. The important feature of the system was that patients could use the unaffected sides to control the affected sides to practice mirror-image movement by a bimanual position feedback strategy. Colombo et al. designed a wrist manipulator with one-degree of freedom and an elbow-shoulder manipulator with two degrees of freedom for the rehabilitation of upper limb movements using admittance control. The admittance control could facilitate the movement with providing target position, velocity, and acceleration based on interactive torque [6]. Recent studies show that mechanical help from robotic system should better not be conducted in a passive mode, and ‘assist-as-needed’ help to promote brain reorganization [7,8].

EMG signals reflect the activities of the muscles, and generate before the formation of muscle force, therefore they are often used to represent the subject’s intention and trigger the robots in prosthesis control [9,10], and robot-aided rehabilitation [11]. Recently, many researchers used EMG signals to continuously control exoskeleton-type robots. These robots were designed like human’s joints and could be worn by the human operators as an assistive devices. The systems were under the voluntary control, functioning like additional muscle groups to provide additional forces [12-16]. However, the robotic system developed by Rosen et al. was applied to share the loading for normal subjects [12,13], and Cheng et al. investigated the movement performance when subjects after stroke were assisted by the system within their voluntary range of motion [14]. The therapeutic effect of myoelectric control during rehabilitation training have not been reported in these studied. Our previous studies had reported the therapeutic effect of myoelectric control to restore motor function of affected elbow [15,16]. The effect of myoelectric control on the sensorimotor control and the therapeutic effect during rehabilitation training of the affected wrists for stroke survivors are not well investigated. In this study, we designed the myoelectrically controlled robotic system and evaluated its effects on joint movement and muscle groups of the paretic side, moreover, the therapeutic effect during robot-aided rehabilitation training was also investigated in terms of clinical scales and robot measured parameters.

## Methods

### Hardware

**Figure 1:**
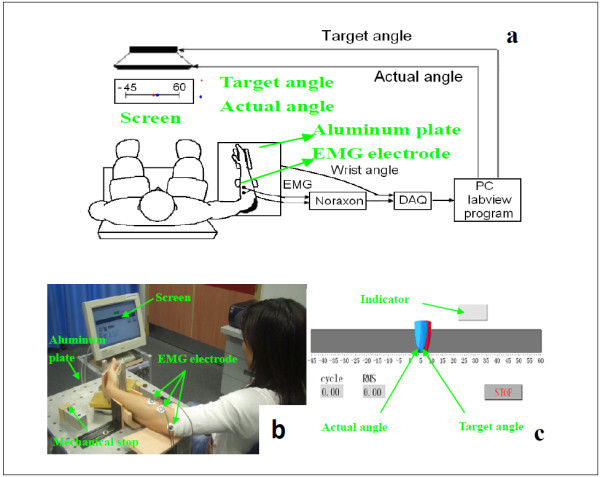
Experimental setup of the myoelectrically controlled wrist robot; (a) block diagram; (b) experimental setup with a stroke subject; (c) Labview interface for the robot-aided rehabilitation training.

A one degree-of-freedom (DOF) robotic system was designed to provide mechanical torque during wrist flexion and extension (Figure 1). The system was consisted of a personal computer (PC), a data acquisition device (PCI 6036E, National instrument, Texas, USA), an actuated mechanical part, and an EMG amplifier. The actuated mechanical part was composed of an aluminum splint, a torque sensor (AKC-205, the 701st Institute of China Aerospace Science and Technology Corporation, China), and a direct drive (DDR) brushless AC servo motor (DM 1045B, YOKOGAWA, Japan), which were connected and rotated with the same axis of rotation. Subjects’ palms were placed inside the splint and straps were used to fix the forearm. The torque sensor can measure the interaction torque between the subject and the system. There was an optical incremental shaft encoder attached into the motor shaft for measuring the joint angle. The EMG signals were captured through EMG electrode (Noraxon, Scottsdale, USA) and amplified by the EMG amplifier. The torque, EMG, and angle signals were captured at 1000 Hz through the data acquisition card into the computer for further analysis. After being processed, the control signal was generated and sent to the servo motor, mechanical help was provided by the motor for assisting the movement of wrist flexion and extension. There was a software program which provided visual feedback of both the target and actual wrist joint angle in a computer screen placed in front of the subject.

### EMG control model

The EMG signals from flexor muscle (FCR) and extensor muscle (ECR) of the affected wrist were used for the proportional control of the mechanical assistive torque during wrist flexion and extension respectively. The raw EMG signals of ECR and FCR were amplified with a gain of 2000 with a band-pass filtered in 10–400 Hz. Then the EMG signals were full-wave rectified and calculated with a moving window (100 ms). The processed EMG signals w, were then normalized to the range 0–1 for NEMG, as follows:

(1) $$\mathrm{NEM}G_{j}=\frac{w_{j}-w_{r}}{w_{\mathrm{mvc}}-w_{r}}$$

where w, was the amplitude of processed EMG signal at rest, and w _mvc_ was maximal amplitude of the processed FCR EMG signal during the maximum isometric voluntary flexion (MIVF) and maximal amplitude of the processed ECR EMG signal during maximum isometric voluntary extension (MIVE) at neutral position. The assistive torque T_assist_ was estimated based on the normalized EMG signals as follows:

(2) $$T_{\mathrm{assist}}=G*T_{\mathrm{mvc}}*\mathrm{NEM}G_{j}$$

where G was the gain for EMG to torque. The EMG-torque gain was set at 0%, 50%, 100%, and 150% in this study. The resultant torque T_res_ the motor could generate is shown in the following equation:

(3) $$T_{\mathrm{res}}=T_{\mathrm{assist}}-T_{\mathrm{resist}}$$

where T _resist_ = *λ*T _MVC,_ T_MVC_ was the MIVE torque during wrist extension and the MIVF torque during wrist flexion. *λ* was a coefficient of the resistive torque, and was set at 0% and 20%. G and *λ* were determined in a pilot experiment with several patients after stroke and based on the performance of their movements.

### Experimental protocol

Sixteen subjects after chronic stroke with moderate motor disability on upper limb were recruited (Demographic information is showed in Table 1). The criteria for recruiting the subjects included the following: (1) there should be at least six months after unilateral stroke; (2) the subjects should not have visuospatial, cognitive or attention deficits which would prevent them from following instructions or performing the experimental procedures; (3) the subjects should have measurable EMG signal from FCR and ECR (the processed EMG signal after the moving window should be at least twice SD of the resting EMG signal), and (4) all subjects did not have any anitspastic drugs after stroke. This study was approved by the human ethical committee of the Hong Kong Polytechnic University. Before the experiment, all the subjects were explained the experimental procedures, and they signed consent forms.

**Table 1:** Demographic information of the 16 stroke subjects; demographic information of the 16 stroke subjects

**[Minimum: Maximum] years after stroke**	**Years after stroke**	**Stroke type Hemorrhage/Ischemic**	**Hemiplegic side left/right**	**Gender Female/Male**	**Age (years)**
[1.5:10]	4.8±5.1	6/10	7/9	4/12	48.2±13.7

All subjects received robot-assisted wrist training, consisting of 20 sessions, 3–5 sessions/week within 5–7 weeks. During each session, all the subjects were instructed to complete two tasks. In the first task, the MIVE and MIVF torques were measured for the affected wrist flexor and extensor when the wrist was positioned at the neutral position. The EMG signals during MIVE and MIVF were also used to normalize the EMG signals of FCR and ECR, and the MIVE and MIVF torques were applied to calculate resistive load in the next stage, respectively. In the second task, subjects were asked to perform a repetitive wrist tracking task with the myoelectrically-controlled system which began with the wrist at 45 deg extension. The subjects tried to control the wrist angle to match the target pointer with this real-time visual feedback from 45 deg extension to 60 deg flexion, and the target would move at a constant speed of 10 deg/s. During wrist extension and flexion, the robotic system generated an assistive torque that was proportional to the amplitude of the processed ECR and FCR EMG signals to assist movement, together with a constant resistive torque that was a percentage of the MIVE and MIVF torque, respectively. The real-time assistive torque and resistive torque provided by the system were based on the equation 1–3. If the subjects could not follow the target pointer, they was suggested to wait for the target pointer to come back so that they could follow it again. There were tasks with 6 different combinations of the EMG-torque gain (50%,100% and 150%) and *λ* (0%, 20%), and subjects were asked to perform each combination twice. Subjects were also asked to perform an evaluation trial twice where the EMG-torque gain and *λ* were equaled to zero. In each session, there were total 14 trials, and each trial consisted 5 cycles of wrist extension and flexion. There was a break of 2 minutes for rest between 2 consecutive trials.

### Data analysis

The torque and angle signals were low-pass filtered using a fourth-order zero-phase Butterworth digital filter with a cutoff frequency of 10 Hz, because the majority of the power of the signals was less than 10 Hz from spectral analysis. Clinical scales were applied to evaluate the subjects before and after the robot-aided rehabilitation training by the same physical therapist who was blinded from the study protocol. These scales included the Fugl-Meyer scale (range 0–66) [17] and was further derived into shoulder/elbow (42/66) and wrist/hand (24/66) subscales [18] for the evaluation of motor function and the modified Ashworth scale (range 0–4) [19] for the muscle tone at the wrist joint. In each session, the MIVF and MIVE torques were used to reflect muscle strength; the active range of motion and accuracy in the evaluation trial of each session can be measured, which can also be applied to evaluate the motor function improvement [15]. The indicator of tracking accuracy can be calculated by the root mean squared error (RMSE) between the actual elbow angle and the target angle. Minimal clinically important difference (MCID) was applied to describe if the training-induced change in clinical scales had clinical significance. On the basis of clinical experience and estimates reported for similar outcome measures in related literature, the MCID was set at 10% of the total range of the scales (6.6 points for the Fugl-Meyer scale and 0.4 point for the modified Ashworth scale) [20]. One-way ANOVA with repeated measures was applied to verify the statistical significance of change in the range of motion and EMG amplitude among different combinations of EMG-torque assistive gain and resistant torque. The paired t-test was used to statistically compare the above-mentioned parameters between pre- and post- 20-session training. The significant level was set at 0.05. All statistical work was performed with SPSS 14 (SPSS Inc., Chicago, Illinois, USA).

## Results

**Figure 2:**
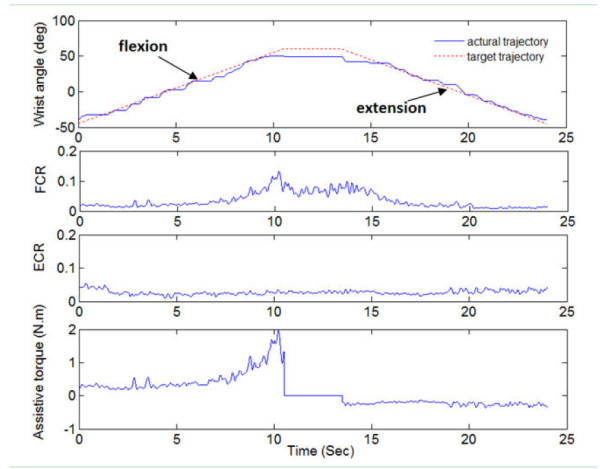
**Trajectories, EMG signals and assistive torque in a cycle; the target trajectory(red dotted line), actual trajectory (blue solid line), normalized EMG signal of ECR and FCR and assistive torque provided by the robot during one cycle when the EMG-torque gain was 50%.** The negative value of assistive torque was the assistive torque during wrist extension; and the positive value was the assistive torque during wrist flexion. There was no assistive torque during the rest time when the wrist angle reached the 60 degree wrist flexion at the middle of this trial.

**Figure 3:**
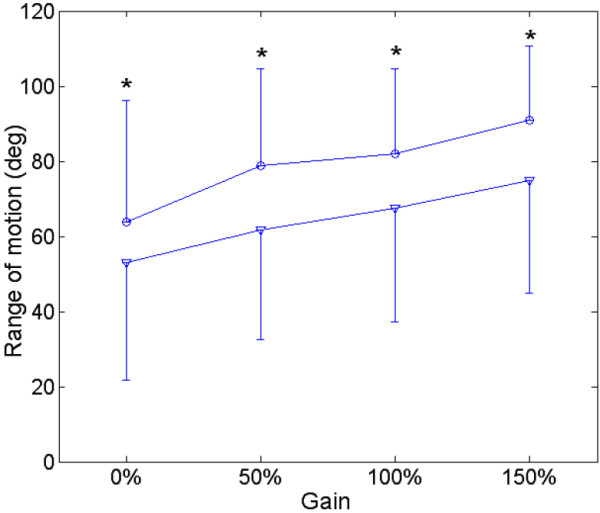
**The range of motion in the first and last training sessions; the range of motion of the wrist movement with different EMG-torque gains from the 16 stroke survivors in the first and last training sessions.** Vertical bars represent one standard deviation. (*: p < 0.05).

**Figure 4:**
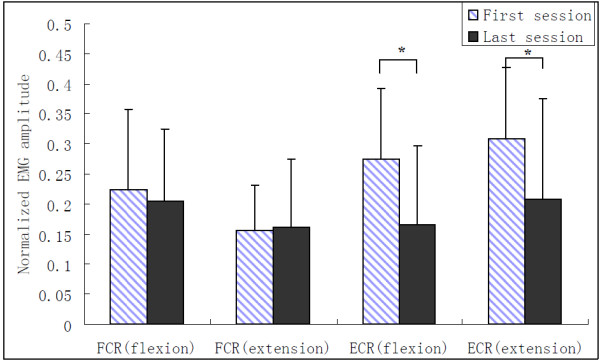
**EMG amplitude in the evaluation trial of the first and last sessions; group mean normalized EMG amplitude of FCR and ECR during wrist flexion and wrist extension in the evaluation trial of the first and last sessions.** Vertical bars represent one standard deviation. (*: p < 0.05).

A typical trajectory of a subject together with the processed EMG signals of FCR and ECR was displayed in Figure 2 when the EMG-torque gain was equaled to 50% and resistant load was equaled to 0%. The group mean range of motion of all the subjects at different EMG-torque gains in the first and last sessions was shown in Figure 3. There was a significant increase in the range of motion for all 16 subjects with the assistance of the myoelectrically controlled robotic system (assistive gain, G = 50%, 100%, and 150%), compared to that without the assistance of the system in the first session shown in Figure 3 (p = 0.014, 0.001 and 0.001, respectively). Besides, there was also a significant increase in the range of motion when the EMG-torque gain increased from 50% to 100% (p = 0.009), and from 100% to 150% (p = 0.003). Stroke survivors could practice movements on their wrist to the unreached range with their voluntary assistance from the robotic system. In the last session, the ranges of motion at the gain of 0%, 50%, 100% and 150% were significant larger than those in the first session (p = 0.036, 0.002, 0.019, and 0.004, respectively). Figure 4 compared the normalized EMG signals in the evaluation trial of the first and last session from all 16 subjects. EMG level of ECR for both wrist flexion and extension in the evaluation trial decreased significantly after the 20-session training (p = 0.011 and 0.019, respectively),while no significant difference was found in the EMG level of FCR (p = 0.65 and 0.88, respectively).

**Figure 5:**
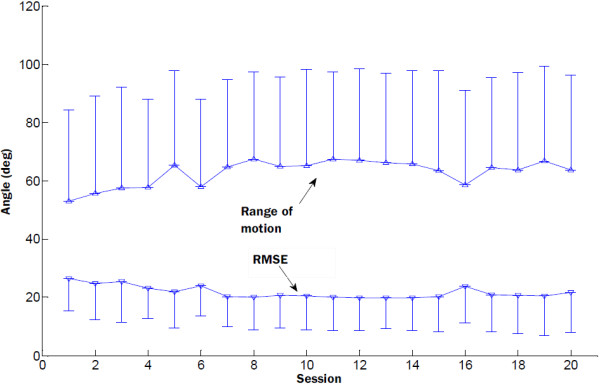
Group mean RMSE and the range of motion; group mean RMSE and the range of motion of all subjects in the 20 training sessions. Vertical bars represent the standard deviation.

**Figure 6:**
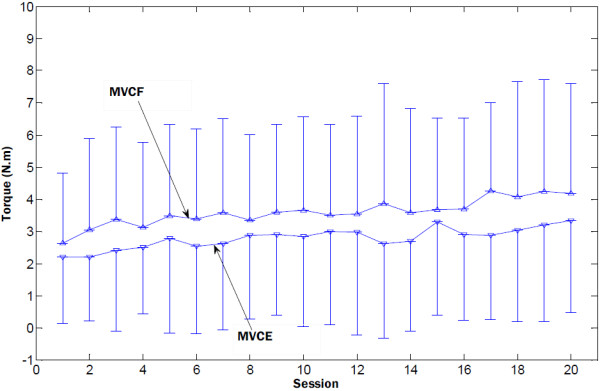
Group mean MIVF and MIVE of all subjects in the 20 training sessions; group mean MIVF and MIVE of all subjects in the 20 training sessions. Vertical bars represent the standard deviation.

Figure 5 presented the group mean RMSE and the range of motion in the evaluation trials of the 20 training sessions. The RMSE dropped abruptly during the first eight of the 20 training sessions, and there was less change during the last several sessions. The range of motion increased in the first half of the training sessions, and reached steady state from session 11. In the second half of the training sessions, there was slight decrease in the range of motion. The torque signals measured by the robotic system were also used to evaluate the improvement in muscle strength during the rehabilitation training. The group mean MIVE and MIVF torques of all subjects were shown in Figure 6. The MIVE and MIVF torques continuously increased across the rehabilitation training. The results showed that the continuous assistance of the myoelectrically controlled robotic system with resistive torque had a positive effect in developing muscle strength. Table 2 summarized the mean values, standard deviations of clinical variables and robot-measured parameters, and the value of the comparison between pre- and post- 20-session training. There was a statistically significant decrease in the modified Ashworth score after the 20-session training that reflected the improvement in muscle tone in the affected wrist. There were significant increases in the Fugl-Meyer scale both for shoulder/elbow and for wrist/hand, which reflected an improvement in upper limb motor function. Both the improvements in the Fugl-Meyer (6.7 points) scale and in the modified Ashworth (0.6 point) scale exceeded the MCID. The RMSE between the measured elbow angle and the target angle showed a statistically significant decrease. There were also statistically significant increases in the MIVF and MIVE torques.

**Table 2:** Values of clinical scales and robot-measured variables pre- and post- 20-session training; values of clinical scales and robot-measured variables pre- and post- 20-session training for all subjects after stroke, in which S/E = shoulder and elbow, W/H = wrist and hand ( * , p < 0.05)

**Parameter**	**PRE**	**POST**	**P**
Fugl-Meyer scale (S/E) (0–42)	18.5 ± 4.4	22.8 ± 7.2	0.001*
Fugl-Meyer scale (W/H) (0–24)	10.9 ± 4.1	13.4 ± 5.9	0.002*
Fugl-Meyer scale for upper limb (0–66)	29.4 ± 7.7	36.1 ± 12.3	0.001*
Modified Ashworth scale (0–4)	1.3 ± 0.5	0.7 ± 0.7	0.001*
RMSE (deg)	26.4 ± 11.0	21.8 ± 13.8	0.046*
Range of Motion (deg)	53.0 ± 31.4	63.7 ± 32.5	0.058
MIVF torque (Nm)	2.6 ± 2.2	4.2 ± 3.4	0.013*
MIVE torque (Nm)	2.2 ± 2.1	3.3 ± 2.9	0.002*

## Discussion

It has been well established that EMG amplitude of agonist muscle increases monotonously with the biological muscle force [21], and proportional control of the mechanical assistive torque based on EMG signal can create a natural integration between human and machine. This study introduced the myoelectrically controlled exoskeleton-type rehabilitation robotics, which used residual voluntary EMG signals from the paretic side as command signals to control the mechanical assistance, and we evaluated this design with 16 subjects after stroke. As we knew, the ability of subject after stroke to perform voluntary movement is limited since the interruption of the descending pathway caused by stroke and the immobilization after stroke [22,23]. Ada et al. found that patients after stroke had selective muscle weakness at a shortened position [24]. Muscle cocontraction was also observed in subjects after stroke which reflected the impairment of the ability to selectively activate flexor and extensor [25]. It was harder or even impossible for these patients to reach the same range of motion as normal subjects due to contracture [26], spasticity [27], or muscle weakness [28]. The assistive function of the myoelectrically controlled robotic system could enable subjects after stroke to perform wrist movement in a larger range of motion than through their own voluntary efforts. The stroke survivors could be trained in the unreached range with their voluntary residual EMG on the paretic side, which is very important for rehabilitation training not only due to its physical beneficial effect but also due to its psychological positive motivation. The improvement in the range of motion without losing the voluntary control could motivate the stroke survivors to practice rehabilitation training, and finally resulted in a better recovery of their motor function.

Although passive movements have been reported to have beneficial effects on restoring the upper limb functions, the principle of ‘assist-as-needed’ is preferred in recent research, since it might help to promote brain reorganization and result in a better therapeutic effect. Rehabilitation training with cognitive investment for these subjects after stroke might promote the reorganization in the brain if the subject could keep efferent efforts to their affected arms rather than merely receiving afferent input in the passive training due to the sensorimotor integration theory [29]. Since EMG signal reflects subject’s intention on the movement, it should relate with the cognitive investment. With the myoelectrically controlled mechanical assistance from the system, it had caused a decrease of the EMG amplitude of agonist muscle. In order to keep cognitive investment to a certain level, resistant torque was applied to our system, which caused an increase in the voluntary effort on agonist muscle. The results showed increases in muscle strength of the wrist flexor and extensor, and improvements in the range of motion after the 20-session training program. Training-induced brain reorganization could explain the physiological mechanisms underlying the improvements after active robot-aided rehabilitation training [30].

The activation of EMG between the antagonist and agonist muscle pair around a joint to provide some damping effect to the joint during the movement, which could contribute to movement accuracy in a dynamic motion [31]. However, due to stroke induced damage, abnormal muscle activation patterns were often found in the paretic limb after stroke. The phenomenon reflected that the limb movements were not energy-saving, and it also associated with the degradation of movement accuracy and efficiency [32]. In this study, a better coordinating pattern could be found by the significant decrease of ECR during wrist flexion and extension together with smaller RMSE after the robot-aided rehabilitation training, which demonstrated the improvement in the upper limb motor functions.

Although both the range of motion and cognitive investment are important to stroke rehabilitation, they are seldom considered together. In previous studies, some researchers focused on voluntary control in a limited range of motion, such as CIMT [33], or some studies led the rehabilitation in a passive way with larger range of motion but less cognitive investment [30]. In our system, the range of motion and the level of cognitive investment can be controlled by the two parameters: EMG-torque gain and coefficient of the resistive torque at the same time. The combination of assistive torque and resistive torque in this present design would simulate the interaction with external objects with different resistive torque. While operating the system, the patient could sense the assistant or resistant torque from the system. The human brain could relearn and used their EMG to control the robotic system through the visual feedback. The bi-manu-tracking trainer was developed by Hesse et al., which enabled the bilateral or unilateral practice of forearm pronation/supination and wrist dorsiflexion/volarextension in a preprogrammed passive mode or bimanual mode [34]. Daily therapy of 15 minutes with the arm trainer and 45 minutes with comprehensive rehabilitation program that included individual physiotherapy and occupational therapy on 12 chronic patients after stroke for 3 weeks resulted in a reduction on the modified Ashworth scale and there was no report of the improvement in functional tasks. While in our study, both muscle tone and upper limb motor function had shown significant improvement, which might be due to the application of myoelectric control.

There are some limitations of the current study, the relationship between EMG signal and joint torque is simplified as linear model, which may change especially in dynamic situation or the EMG-force relation may have various and dramatic slope changes after stroke [35], but this simplified model help subject to easily learn how to control the robotic system. With the develop of myoelectric pattern classification of arm movements in the recent research [36,37], subjects after stroke can perform rehabilitation training with a multi-joint myoelectrically controlled robotic system in the next step, and the trajectories in the rehabilitation training can be related to activities of daily living. Moreover, a randomized controlled experiment with a follow up session should be considered in order to further validate the effectiveness of robot-assisted rehabilitation using myoelectric control and compared with passive exercise training.

## Conclusion

Myoelectrically controlled robotic system has been designed to facilitate wrist movement through the stroke survivors’ intention on the affected limb. With the myoelectrically-controlled assistive torque, the stroke survivors could be trained in the unreached range with their voluntary residual EMG on the paretic side. After 20-session rehabilitation training, there were significant improvements in muscle strength and clinical scales. These results indicate that robot-aided therapy with voluntary participation of patient’s paretic motor system using myoelectric control might be a promising method for clinical use.

## Competing interests

The authors declare that they have no competing interests.

## Authors’ contributions

RS, KT and XH designed the study and carried out the experiment. Both KT and RS analyzed the data. KT, RS and WZ interpreted the results, drafted and revised the manuscript. All authors read and approved the final manuscript.

## Authors’ information

Rong Song received the B.S. degree in electrical engineering from Tsinghua University, Beijing, China, in 1999, the M.S. degree in electronic engineering from Shantou University, Shantou, China, in 2002, and the Ph.D. degree in biomedical engineering from the Hong Kong Polytechnic University, Kowloon, Hong Kong, in 2006. He is currently associate professor in school of engineering, Sun Yat-sen University, P.R. China. His research interests include musculoskeletal modeling, biomedical signal processing, human motion analysis, and robot-assisted stroke rehabilitation. Kai-yu Tong received the Ph.D. degree in bioengineering from the University of Strathclyde, Glasgow, U.K., in 1998. He spent four months as a Research Fellow at Strathclyde University and participated in a joint project with the Spinal Cord Injury Unit, Southern General Hospital, Glasgow, U.K. He joined the Hong Kong Polytechnic University in 1999 and currently as a Professor in the Interdisciplinary Division of Biomedical Engineering and Program Leader in Biomedical Engineering. His research interests include rehabilitation robot, the control of functional electrical stimulation for upper and lower extremity functions, sensor development, stroke rat model and gait training rehabilitation on persons after stroke.
